# Special Issue: “Updates on HBV Infection”

**DOI:** 10.3390/microorganisms10030580

**Published:** 2022-03-07

**Authors:** Isabelle Chemin, Flor Helene Pujol

**Affiliations:** 1 Centre de Recherche en Cancérologie de Lyon INSERM U1052, CNRS UMR5286, Université de Lyon, 69003 Lyon, France; 2 Laboratorio de Virología Molecular, Centro de Microbiología y Biología Celular (CMBC), Instituto Venezolano de Investigaciones Científicas (IVIC), Caracas 1020A, Venezuela; fhpujol@gmail.com

Hepatitis B virus (HBV) infection remains a global public health issue: a number of barriers still hamper the control of the HBV epidemic and in finding a cure for HBV [1,2]. The WHO (World Health Organization) estimates that 2 billion individuals (1 person out of 3) have serological evidence of past or present infection and that 257 million people currently live with chronic HBV infection, which is associated with increased liver disease and liver cancer risk [3]. Around 60% of the world’s population lives in areas where HBV infection is highly endemic, including China (total population, 1.3 billion), Indonesia (222 million), Nigeria (132 million), and much of the rest of Asia and Africa. In addition to these high levels of exposure in certain regions around the world, genetic variability within the HBV genome and factors related to its mode of replication in its human host can impact one’s risk of liver disease.

HBV belongs to the family Hepadnaviridae: it is an enveloped virus with a nucleocapsid or core that partially encases double-stranded DNA with only 3200 bases, the viral polymerase, and some host-derived proteins. This virus has a very unique replication cycle, which includes a replicative RNA intermediate molecule, which is why the viral polymerase is a reverse transcriptase for genome replication [4]. Several steps in the replication cycle have been exploited as potential targets for the design of antiviral strategies. Among them, the HBV capsid is a particular element that exhibits many of the features that are essential for trafficking to the nucleus, genome replication, and subsequent morphogenesis [5]. Some of these features are still under investigation [6]. Despite the existence of several interesting targets in the HBV replication cycle, antiviral therapy against HBV is still under development [7].

In addition, HBV can also have a latent mode of infection, called occult infection, where classical serological markers of active infection are absent [8]. Highly sensitive molecular tests are needed to detect these clinical presentations [9].

HBV is mostly a hepatotropic virus but may also infect the cells of the lymphatic system [10,11,12]. It is known that HBV entry into hepatocytes occurs through the binding of the HBV preS1 surface protein to its specific receptor, the bile acid transporter, sodium taurocholate co-transporting polypeptide (NTCP). Despite the fact that the mechanism of HBV entry into lymphatic cells remains unknown, the pre-S1-encoded surface protein is thought to be implicated. Extrahepatic HBV infection has been studied in chronic HBV, and it has been shown that HBV genomes are present in different PBMC subsets from chronically infected carriers. Some studies have shown the presence of HBV variants in PBMC, different from the ones found in the plasma or liver, suggesting HBV compartmentalization. Altogether, HBV infection in PBMCs is implicated in different aspects of HBV pathogenesis, potentially in its persistence, evolution, and possibly in its vertical transmission.

HBV is transmitted by percutaneous or mucosal exposure to infected body fluids (from higher viral concentration to lowest: blood, serum, semen, saliva) and can be divided into two categories [13]: vertical, or mother-to-child or perinatal transmission, and horizontal transmission, which can occur through many different sources of human contact, such as from healthcare occupations, unprotected sex (for instance, men who have sex with men (MSM) and sex workers are at higher risk), or needle-sharing (drug injection, tattooing, acupuncture, etc.), and even via household members or playmates. With such variable sources, horizontal transmission can strike in high- and low-prevalence regions. HBV can remain stable and infectious on environmental surfaces for at least 7 days. Thus, transmission may also take place indirectly via contaminated surfaces and other objects [14].

In fact, the transmission routes vary depending on the prevalence of the geographic region and are probably dependent on the infecting HBV genotype. Genome-wide nucleotide divergence analysis has allowed for the identification of ten HBV genotypes (A–J) and several subgenotypes or subtypes [15] with distinctive distribution patterns and potential clinical associations. The classification is based on the percentage of nucleotide divergence between two HBV sequences. The current global consensus is based on a nucleotide divergence of >7.5% for the definition of distinct genotypes and of 4–7.5% for subgenotypes [16,17]. In Asia, genotypes B and C are known to be more frequently transmitted because mothers generally exhibit a highly replicative and HBeAg-positive infection. In contrast, in Africa, genotype E transmission is almost solely horizontal since the HBeAg seroconversion of the mother occurs at a young age. In low and intermediate endemic countries, transmissions are less exclusive, and exposure routes vary depending on various factors, such as drug usage to healthcare facilities or unprotected sex. Genotypes A–D are the best characterized types, with genotype A being the most frequent in North America, Europe, Southeast Africa, and India. Despite being closer at the genomic level, HBV subgenotypes exhibit very distinct geographical distributions and clinical impacts and deserve further studies [18,19]. With between 4% and 7.5% intergroup nucleotide divergence across the complete genome and good bootstrap support, genotypes A–J can be further organized into almost 40 subgenotypes.

The natural history of chronic hepatitis B virus (HBV) infection and disease is complex and highly variable. Chronic hepatitis B (CHB) and nonalcoholic fatty liver disease are increasingly observed together in clinical practice, and the development of nonalcoholic steatohepatitis (NASH) represents another leading cause of liver-related morbidity and mortality [20,21,22,23]. There is a growing understanding of how viral, host, and environmental factors influence disease progression, which could ultimately improve the management of chronic hepatitis B. In this Special Issue, we try to explore the natural history of chronic hepatitis B and emphasize the factors influencing the course of liver disease.

Finally, as described before, the management of hepatitis B disease is quite a complex matter, and it could benefit from the know-how that has already been accumulated by addressing HIV [24].
